# Single Stance Stability and Proprioceptive Control in Older Adults Living at Home: Gender and Age Differences

**DOI:** 10.1155/2013/561695

**Published:** 2013-07-28

**Authors:** Dario Riva, Carlo Mamo, Mara Fanì, Patrizia Saccavino, Flavio Rocca, Manuel Momenté, Marianna Fratta

**Affiliations:** ^1^International Society of Proprioception and Posture, Turin, Italy; ^2^Proprioception Center, Via Valgioie 85-87, 10146 Turin, Italy; ^3^Epidemiology Unit, Local Health Unit TO3, Piemonte Region, Grugliasco, Italy; ^4^Sanitary District 2, Local Health Unit TO1, Piemonte Region, Turin, Italy; ^5^Department of Functional Rehabilitation, Local Health Unit TO1, Piemonte Region, Turin, Italy

## Abstract

In developed countries, falls in older people represent a rising problem. As effective prevention should start before the risk becomes evident, an early predictor is needed. Single stance instability would appear as a major risk factor. Aims of the study were to describe single stance stability, its sensory components, and their correlation with age and gender. A random sample of 597 older adults (319 men, 278 women) living at home, aged 65–84, was studied. Stability tests were performed with an electronic postural station. The single stance test showed the impairment of single stance stability in older individuals (75–84 yrs). The significant decline of stability in the older subjects may be explained by the impairment of proprioceptive control together with the decrease in compensatory visual stabilization and emergency responses. Younger subjects (65–74 yrs) exhibited better, but still inadequate, proprioceptive control with compensatory visual stabilization. Gender differences appeared in older subjects: women were significantly less stable than men. The measurement of the sensory components of single stance stability could aid in the early detection of a decay in antigravity movements many years before the risk of falling becomes evident. Adequate proprioceptive control could mitigate the effects of all other risks of falling.

## 1. Introduction

Unstable balance, falls, and loss of independence in the older population are a growing problem in the developed countries [1, 2]. The safety of walking and balance are indispensable requirements to prevent falls and loss of independence [3–6]. 

Safe mobility is primarily based on the integration of the proprioceptive, visual, and vestibular inputs [7–11].

Afferent proprioceptive inputs are conveyed to different levels of the central nervous system [10, 12, 13], but most remain unconscious and only a very few (approximately one signal out of a million) are able to reach the conscious level [14]. The joint position sense and the joint movement sense (kinesthesia) are the expression of the conscious component, while postural control is mainly based on the unconscious component [13]. In the case of the antigravity movements, proprioceptive control is the expression of the effectiveness of the stabilizing reflexes in controlling vertical stability [12]. By antigravity movements, we mean activities which require the individual to counteract gravity and postural instability with at least a phase of single-limb stance (walking, running, jumping, going up and down stairs, and so forth). Proprioceptive inputs are the most important sensory system in the maintenance of static postural stability at all ages [8, 15].

Several studies have shown that impaired vision reduces postural stability and increases the risk of falling in older people [16–19]. Consequently, maximizing vision is an effective strategy for preventing falls [20]. Other studies have shown that visual field dependence as postural stabilizer is a risk factor [21, 22]. This visual dependence may develop in response to impaired proprioceptive and vestibular systems as a result of age and chronic health problems [21, 22]. Beyond age 65, the contribution made by vision to balance control declines [8].

The role of the vestibular inputs in maintaining postural stability has been investigated mainly in double stance [7], and most studies have shown age-related differences [8, 15]. In order to isolate the function of the vestibular signals, the stability tests were performed excluding the visual information and diminishing the proprioceptive inputs in different ways, for example, by changing the inclination of the base of support [7, 15] or using different compliant surfaces [8]. In both cases the proprioceptive inputs cannot be abolished, and the decrease in the proprioceptive involvement cannot be quantified. Moreover the proprioceptive mitigation could limit the expression of the vestibular responses [8]. While the visual inputs can be excluded without affecting the motor responses based on proprioceptive and vestibular inputs, on the contrary proprioceptive and vestibular inputs cannot be isolated without compromising the responses based on the remaining signals. For these reasons in the measurement of proprioceptive control it could be more useful to include the possible intervention of the vestibular responses in order not to compromise the expression of the proprioceptive potential. Similarly, it might be preferable to assess the full potential of the emergency responses without diminishing the proprioceptive inputs and considering instead how the joint action of the proprioceptive and vestibular systems is able to face an increase in instability.

The fact that the single-limb support period accounts for 80% of the gait cycle at normal walking speed, while the double-support period accounts for 20% [23–25] suggests an important role for single stance stability in the safety of walking. Studies have reported that increasing age is associated with decreased stride length, speed, single support time, and increased stride width [26–29]. However, other studies have reported that aging is not the primary factor for decline in gait parameters [29–31]. Decreased stride length, speed, and single stance phase may in fact be stabilizing adaptations related to fear of falling [9, 26, 29, 30, 32].

For the above reasons, single stance stability could be the key element of the effectiveness and safety of antigravity movements, and the very refined single stance stability of world and Olympic champions [33] enforces this hypothesis. Postural stability has been measured in different sensory conditions (with eyes open and closed, on firm and compliant surfaces) and in different stances (double or single). Age-associated worsening in postural stability and sensori-motor functions has been shown in older people in double stance [8, 15, 34–37]. In single stance, age-related impairment of postural stability has also been shown, but sensori-motor functions were not investigated [35, 38–40]. Gender-related differences are more controversial: some studies in double stance have shown no differences in postural sway [36, 37] while other authors have found that postural sway is higher in women at all ages [34] or under stressing balance conditions [41]. In single stance, gender differences have been investigated only with timed tests and in a few studies: one author has found that men are more stable than women at all ages [38] while a more recent study has shown no differences between genders [40].

The heterogeneity of the study methods and the limited sample size entail that the reported data in literature do not clarify the measure of the decay of single stance stability and of its sensory components associated to aging and gender.

The aims of the present study were to describe, by means of an instrumented test, the characteristics of single stance stability and its proprioceptive and visual components in a representative sample of older adults and their correlation with age and gender. Additionally, single stance stability was compared to double stance stability.

## 2. Methods

### 2.1. Study Subjects

A random sample of older men and women resident in Turin (an industrial town in North-western Italy) was recruited by means of a sex-stratified random sampling from the general population. The database of the Turin Longitudinal Study (which includes the whole resident population of Turin) was used as the data source for the sampling [42]. A letter of invitation, presenting the objectives of the study, was sent to 750 men and 750 women in the age class 65–84, resident in the Second Administrative District of the Local Health Unit TO1. It was possible to contact 688 men and 703 women by phone to establish a date for performing the tests. 16 men and 39 women did not meet the inclusion criteria during the phone call. 374 men and 324 women accepted to participate (298 men and 340 women were not interested). 41 men and 37 women did not attend the appointment; 333 men and 287 women were tested, with a response rate of 89%. All subjects signed an informed consent form that summarized the purpose of the study, explained risks and discomforts, and indicated that all information gathered would remain confidential. Prior to the tests, a medical history questionnaire was administered to collect data about the subject's health status. In order to be included in the study, subjects had to be able to walk for at least six meters without an assistive device and to perform the tests. Subjects with cognitive deficits or medical problems were excluded only if the condition prevented them from meeting these inclusion criteria. 14 men and 9 women were excluded from the study because they did not meet the inclusion criteria at the moment of the questionnaire/tests or because their data were not complete. In total, 597 adults (319 men, 278 women), living at home, were included in the study and analysis. Tests and interviews were performed between April and September, 2010. Prior to the study, a pilot study was conducted on 20 subjects in order to check the feasibility and to improve the design of the research. The subjects in the pilot study were not included in the analysis.

### 2.2. Postural Stability Assessment

#### 2.2.1. Instruments

**Figure 1:**
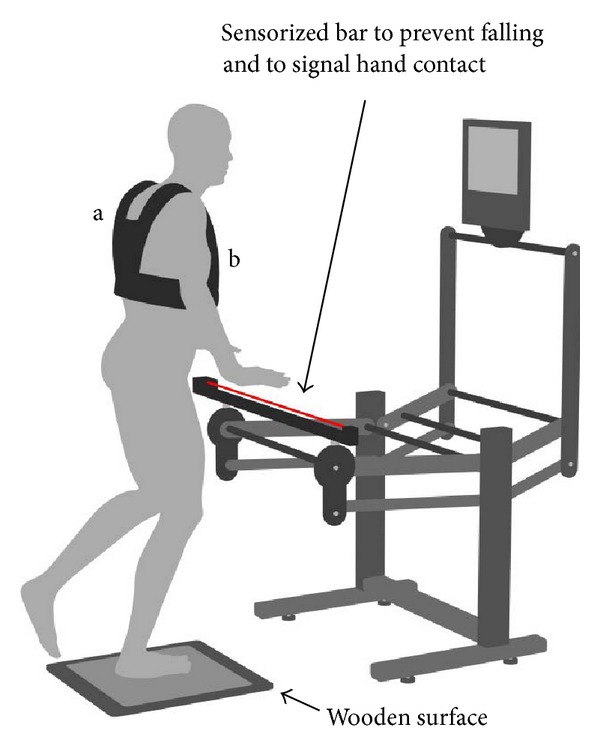
The postural proprioceptive station. The red line represents the infrared ray of the sensorized bar. Vest (a) to support the “postural reader”, (b) a two-dimensional accelerometer unit, in sternal position.

The stability tests were performed by means of an electronic postural proprioceptive station (DPPS, Delos, Turin, Italy) [43] connected to a personal computer with a specific software (DPPS 5.0). In Figure 1, the station included an electronic postural reader (DVC, Delos Vertical Controller), a sensorized bar, and a display. The DVC, applied to the sternum, measured the trunk inclination in the frontal (*x*) and sagittal (*y*) plane by means of a two-dimensional accelerometer unit. The rotational radius of the DVC, when applied to the sternum, in most cases lays between 0 and 15 centimeters, never exceeding 30 centimeters. In fact, the trunk of a subject in single stance moves as a segment of a broken line with multiple joints. To minimize the fall risk, subjects could lean on the horizontal bar placed in front of them, at an adjustable height, to regain vertical control rapidly. The bar was equipped with an infrared sensor able to indicate when subjects leant on it.

#### 2.2.2. Algorithms

The data from the postural reader are a stream of acceleration samples taken by converting into digital domain the sensor outputs, at a rate of 100 Hz. These raw data were initially averaged with a 4-tap sliding window, so the 3 dB bandwidth was narrowed to about 11 Hz. A scaling with the calibration data of the DVC was then performed, and an arcsin function was applied to convert the raw data into angles:

(1) $$\begin{array}{c} \alpha \left(i\right)=\arcsin\left[2\left(\frac{dx\left(i\right)-dx_{-90}}{dx_{+90}-dx_{-90}}-0.5\right)\right], \end{array}$$

(2) $$\begin{array}{c} \beta \left(i\right)=\arcsin\left[2\left(\frac{dy\left(i\right)-dy_{-90}}{dy_{+90}-dy_{-90}}-0.5\right)\right], \end{array}$$

where *dx*(*i*) and *dy*(*i*) are the generic elements of the raw data stream while those with the numerical indexes are calibration data taken at the mechanical limits of the instrument. Due to the nature of the sensor, the raw data are affected by linear accelerations superimposed on the inclination information. The measurement error is mainly proportional to the rotational speed and radius, momentarily counteracting the real movement; in static conditions it disappears. As the disturbance is mainly located at high frequencies, a pole at about 1.1 Hz was digitally applied to the data stream in order to get a good approximation of the position; the validity of this assumption was confirmed by numerical simulation and video recording compared to the system output. In the following parts of this paper, the filter outputs will be taken as angle samples. Each of them had a contact attribute according to the presence or absence of hand contact with the sensorized bar. The postural assessment was based on two components: autonomy (*Au*) and the average postural instability (*PIxy*). *Au* is the percentage of the trial without hand contact and was calculated as

(3) $$\begin{array}{c} Au=100\frac{n-n_{c}-n_{m}}{n}, \end{array}$$

where *n* is the total number of samples, *n*_*c*_ the number of samples with contact attribute, and *n*_*m*_ a correction amount that will be defined hereafter.

As each hand contact with the sensorized bar has a stabilizing posteffect, consequently multiple very short hand contacts could significantly overestimate the actual value of *Au*. In order to consider this effect, an additional time (malus time) was applied to each contact period with duration between 0.06 and about 2 seconds. This operation was performed by forcing the contact attribute of a certain number of samples, starting from the end of the considered contact. In case of a further contact, this action was stopped, and the contact time measurement was also restarted in order to eventually apply a correction after this new contact. *Au* was calculated on the basis of the corrected contact data set. The method of calculating the correction amount (*m*) was defined empirically, with an inverse relation to the length of the contact period. This correction ranged from a maximum of 0.674 s (for the shortest considered contact period of 0.060 s) to zero (for hand contact periods greater than 2.02 s). If the *j*th contact has a duration *t*_*c*_(*j*),  the correction should last (only positive corrections are applied):

(4) $$\begin{array}{c} m\left(j\right)=\frac{\log\left(\text{MM}/t_{c}\left(j\right)-1\right)\;+\text{SF}}{\text{TF}}, \end{array}$$

where MM = 3.3 s is the malus maximum factor, *t*_*c*_(*j*) is the duration of a contact event in seconds, SF = 0.2 is the shift factor,  and TF= 2.8 Hz is the twist factor.

Being *Fs* = 100 Hz the sampling rate, the number of samples with forced contact attribute following the end of the *j*th contact event will be *n*_*e*_(*j*) = *Fs* · *m*(*j*) or *n*_*e*_(*j*) < *Fs* · *m*(*j*) in case of a new contact interrupting the forced action.

The total number of samples with forced contact attribute will then be

(5) $$\begin{array}{c} n_{m}=\sum_{j=1}^{C}n_{e}\left(j\right), \end{array}$$

where *C* is the total number of contact events.

The second component of the postural assessment is *PIxy*, which derives from the average instability in the frontal and sagittal planes (*PIx*, *PIy*). *PIxy* is an indicator of the average radius of the postural cone of instability. *PIx, PIy,* and *PIxy* are expressed in degrees. *PIx* was calculated as the average of the absolute shifting around the average position *α*_*a*_:

(6) $$\begin{array}{c} PIx=\frac{1}{n}\sum_{i=1}^{n}\left|\alpha \left(i\right)-\alpha_{a}\right|. \end{array}$$

The *n* elements of data vector *α*(*i*) were averaged in order to calculate *α*_*a*_:

(7) $$\begin{array}{c} \alpha_{a}=\frac{1}{n}\sum_{i=1}^{n}\alpha \left(i\right). \end{array}$$

*β*_*a*_ and *PIy* were calculated in the same way as *α*_*a*_ and *PIx*. 

The absolute instant deviation from the average axis (*dDVC*(*i*)) was determined as

(8) $$\begin{array}{c} dDVC\left(i\right)=\sqrt{{\left(\alpha \left(i\right)\;-\alpha_{a}\right)}^{2}+{\left(\beta \left(i\right)-\beta_{a}\right)}^{2}.} \end{array}$$

*PIxy* was calculated as

(9) $$\begin{array}{c} PIxy=\frac{1}{n}\sum_{i=1}^{n}dDVC\left(i\right). \end{array}$$

*Stability Index.* A suitable index for ranking all kinds of performances from the highest (very narrow cone with complete autonomy) to the lowest level (very low autonomy) was needed, capable of classifying the performances in the transition zone where both *Au* and *PIxy* decrease. To overcome the limits of *Au* and *PIxy* in describing stability performances, it is necessary to consider the real contribution of each single sample. *A* sample performance value (*spv*(*i*)) was introduced by weighting the *dDVC* (8) according to its value and its contact attribute:

(10) without  hand  contact  {spv(i)=10−dDVC(i)/25(dDVC(i)<50°)spv(i)=0.01(dDVC(i)≥50°),

(11) with  hand  contact  {spv(i)=0(dDVC(i)<2°)spv(i)=10(dDVC(i)−32)/10+0.0001(dDVC(i)  −12)(2°≤dDVC(i)≤12°)spv(i)=0.01(dDVC(i)>12°).

The different thresholds were defined after experiences in the field. They ensure that without contact *spv* is always higher than when contact is present. In the case of contact when *dDVC* is less than 2°, *spv* equals 0, because it means that the subject is unable to widen the postural cone by activating the emergency responses (proprioceptive or vestibular). In fact the appropriate action to counteract a sudden risk of fall should be to break the vertical line of the body at hip level instead of relying on an immediate hand support. For *dDVC* between 2° and 12°, *spv* rises as *dDVC* grows, to reward the attempt to recover before using hand contact; then it saturates at 0.01. The sum of all *spv* gives the result of the whole trial. In the case of a virtually perfect trial (a standstill trial without hand contact, where also reading errors and measurement noise are nulled) each *dDVC* equals 0 and each *spv* equals 1. Consequently the maximum possible *spv* sum of a trial will equal the number of samples. The stability index (*SI*) is the relation (in %) between the *spv* sum of the present trial and a virtually perfect one:

(12) $$\begin{array}{c} SI=\frac{100}{n}\sum_{i=1}^{n}spv\left(i\right), \end{array}$$

where *spv*(*i*) is the *spv* of the *i*th sample and *n* is the total number of samples. *SI* is a scalar number which makes it possible to rank different trials on a functional scale.

#### 2.2.3. Double and Single Stance Tests

Double and single stance stability was tested with eyes open (EO) and closed (EC). The subject, barefooted, was asked to minimize *PIxy* while staying in double or single stance on a stable wooden surface. The subject was looking at a display which showed the countdown before each trial, the eye condition requested, and in single stance which foot was to be used as support. No feedback on postural stability was given during the tests. The sequence proceeded automatically. Each trial lasted 20 seconds followed by a pause of 15 seconds. A red rectangle appeared on the display when the subject touched the sensorized bar: this warning urged the subject to touch the bar only when absolutely necessary to prevent falling and as briefly as possible. The double stance test (DT), with feet as close together as possible, consisted of 2 trials with eyes open and closed. It was performed twice: DT1 and DT2 (four trials in total). The single stance test (ST) consisted of four trials. Each leg performed a first trial with EO and a second trial with EC, in an alternated sequence of the left and the right limbs. It was performed twice: ST1 and ST2 (eight trials in total). The subject stood with the weight-bearing knee bent to 170° and the non-weight-bearing knee flexed to 45° (Figure 1). Prior to the ST, participants were provided with a propaedeutic test of shorter duration to familiarize with single stance. The average of the two limbs for all variables of ST was considered.

#### 2.2.4. Basic Variables

*Au, SI, *and *PIxy* were the basic variables considered to analyze the eight trials of ST and the four trials of DT. The precautionary strategy (*Ps*) was represented by the complementary value of *Au* (100−*Au*). 

#### 2.2.5. Indicators of Proprioceptive Control and Emergency Responses in ST

The stability index of EO trials was taken as an indicator of postural control (all sensory channels open) while the stability index of EC trials was considered an indicator of proprioceptive control and of its effectiveness as primary stabilizer. High values of stability index in EC trials correspond to refined proprioceptive control (narrow cones), because they are the expression of effective proprioceptive reflexes able to rapidly stabilize the subject before the vestibular responses can be activated [44]. Intermediate values of stability index in EC trials may include the intervention of the vestibular system, but they are in any case the expression of rougher proprioceptive control. In fact, as the vestibular system has a higher threshold of intervention and takes longer before becoming active [45], it can intervene only if proprioceptive control is not refined and therefore permits a longer period for activation [44]. Low levels of stability index are characterized by a progressive increase in precautionary strategy and the decreasing probability of intervention of the vestibular responses.

The emergency responses are the compensatory counter-movements which permit the management of a rougher vertical control without the intervention of the precautionary strategy. They are under the control of the vestibular and proprioceptive systems. The widening of *PIxy* to face a lack of stability was considered an indicator of the capability of activating the emergency responses.

#### 2.2.6. Indicator of Visual Gain and Visual Dependence

The difference between EO and EC trials quantified the visual gain and the relative visual dependence of postural stability. Visual gain and visual dependence are two different ways of describing the same thing: the first quantifies the increase in stability due to the intervention of vision, and the latter quantifies the loss of stability due to the exclusion of vision.

### 2.3. Statistical Analysis

As it is known that the physical performance of older men differs from that of women, analyses were conducted separately for men and women [46, 47]. The means and the standard deviations of every test were measured, stratifying by sex and by age class within each gender. The *t*-test was used for comparing the performance of different subgroups of subjects (males versus females, 65–74 years versus 75–84 within each gender). The values of *P* were two tailed, and *P* ≤ 0.05 was considered to indicate statistical significance. All analyses were performed using the SAS statistical software (version 9.2; SAS Institute Inc., Cary, NC, USA). 

### 2.4. Test-Retest Reliability

In order to assess the test-retest reliability, each test was performed twice and the Pearson's correlation coefficient (*r*) was calculated [48]. When the variable showed a value greater than 0.80, the first trial of the test was considered. If the value was lower than 0.80, the better result of the two trials was chosen. For ST, in EO condition, the average between the left and right trials of the first test was considered in relation to its high test-retest reliability (ST1 versus ST2: *r* = 0.86). In EC the moderate test-retest reliability of the average between the left and right legs (ST1 versus ST2: *r* = 0.76) prompted the choice for each limb of the better trial between ST1 and ST2. The best trial, rather than the average between ST1 and ST2, is considered to express the real functional level of the subject, just as the value of an athlete is described by his/her best result (of an entire career, of a season). For DT the test-retest reliability was low in EO condition (DT1 versus DT2: *r* = 0.38) and moderate in EC condition (*r* = 0.69). Consequently in both conditions the better of the two trials was considered. 

## 3. Results

The characteristics of the 597 participants in this study (319 males, 278 females) are summarized in Table 1. Since the health of Turin's population is related to socioeconomic factors [20], we tested whether the social class of the recruited subjects might have influenced the generalizability of the results: as would be expected, the educational level of respondents was similar to that of nonrespondents.

*Single Stance Test.* The results of ST are shown in Tables 2 and 3. Considering the eight trials of ST1 and ST2, the basic variables (*SI*, *Au* and its complementary value *Ps*, *PIxy*) were calculated from the following combinations of trials: the average between the two limbs of EO trials in ST1 and the average between the two limbs of the better EC trial for each limb in ST1 and ST2. Consequently each variable is identified by a compound name which includes the eye condition and the name of the basic variable. The *Au *and *SI* variables within each combination all showed a correlation coefficient (Pearson) greater than 0.97.

**Figure 2:**
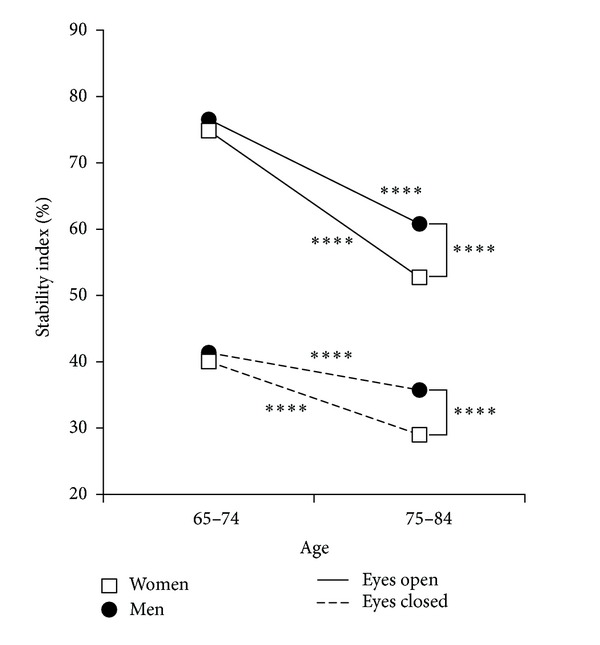
Single stance test: Stability index in men and women according to age. Values of stability index in eyes closed (indicator of proprioceptive control) and eyes open (all sensory channels open) conditions; ****P < 0.001.

*Age and Gender Relations (Table 2).* All variables related to single stance stability were significantly better in the age group 65–74 years both for men (*P* < 0.005) and for women (*P* < 0.001). In the following parts of this paper, if not specified otherwise,* SI* will be the basic variable considered. Both performances in EO and EC showed that the differences between sexes were not significant in the younger subjects (Figure 2) but became highly significant in the older individuals (EO_*SI*: *w* = 52.7 versus *m* = 60.8, *P* < 0.001; EC_*SI*: *w* = 29.0 versus *m* = 35.7, *P* < 0.001). The EO performance decreased from 74.9 in younger women to 52.7 in older women (*P* < 0.001). In males the values were, respectively, 76.6 and 60.8 (*P* < 0.001). Among the younger subjects, 45.4% of males and 36.8% of females were able to perform the tests with EO on each leg without hand support to prevent fall. In the older individuals, the percentage dropped to 16.5% for males and 11.2% for females. 

**Figure 3:**
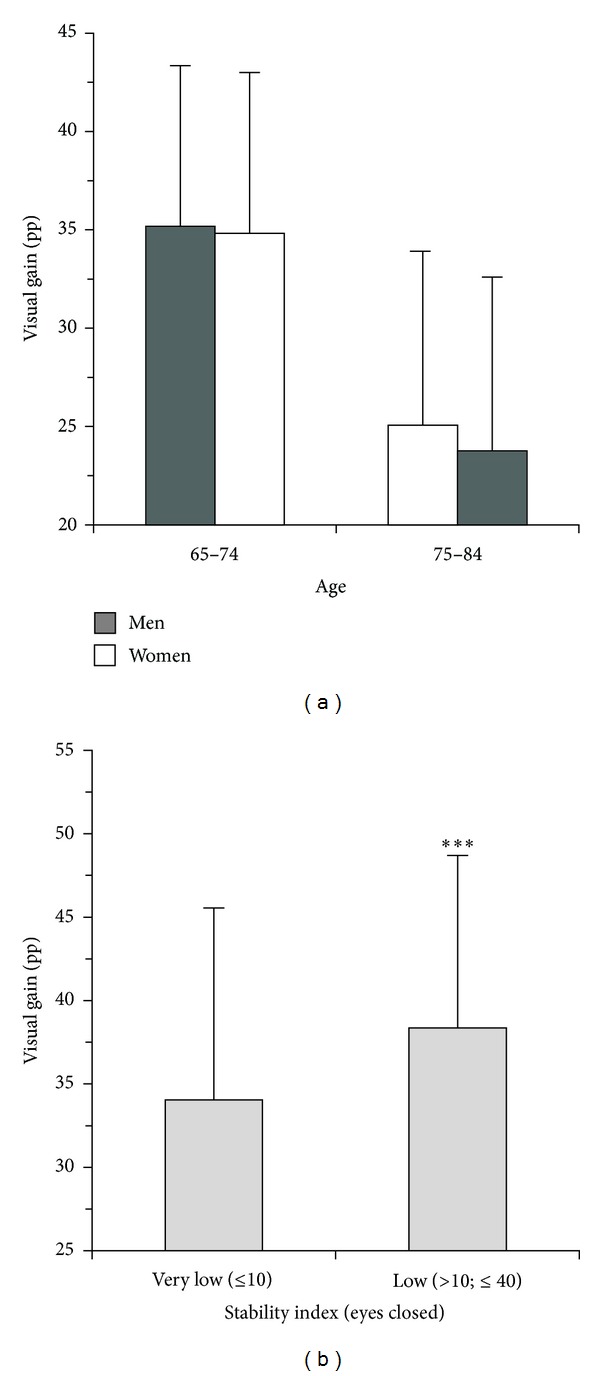
Single stance test (ST). The visual gain in supporting stability. (a) Visual gain according to sex and age: older women and older men showed a similar significant decrement of visual gain (*P* < 0.001) without gender differences. The greater decline of single stance stability in older women with respect to older men would be due to a more accentuated loss of proprioceptive control. (b) Visual gain according to different ranges of stability index in eyes closed; pp: Percentage points; ***P < 0.005.

*Visual Gain and Visual Dependence (Table 3).* The difference between EO and EC trials quantified the visual gain and the relative visual dependence of postural stability. The visual gain and visual dependence in the younger subjects were high and similar for both sexes [(EO-EC)_*SI*: *m* = 35.2, pp; *w* = 34.9 pp; ns]. They decreased, instead, significantly in the older subjects, both in males (−10.1 pp, *P* < 0.001) and in females (−11.2 pp, *P* < 0.001), without significant gender differences (Figure 3(a)). To investigate this trend, we analyzed the visual gain of all ST. The visual gain of the tests where the EC trials showed a very low stability (*SI* ≤ 10) was compared to the visual gain of the tests where the EC trials showed a less low stability (10 < *SI* ≤ 40). The trials were selected considering both EC trials of each leg in ST1 and ST2. The visual gain (Figure 3(b)) was significantly lower in the case of very low EC stability (mean 34.1 ± 25.9 versus 38.4 ± 21.7; *P* < 0.005).

**Figure 4:**
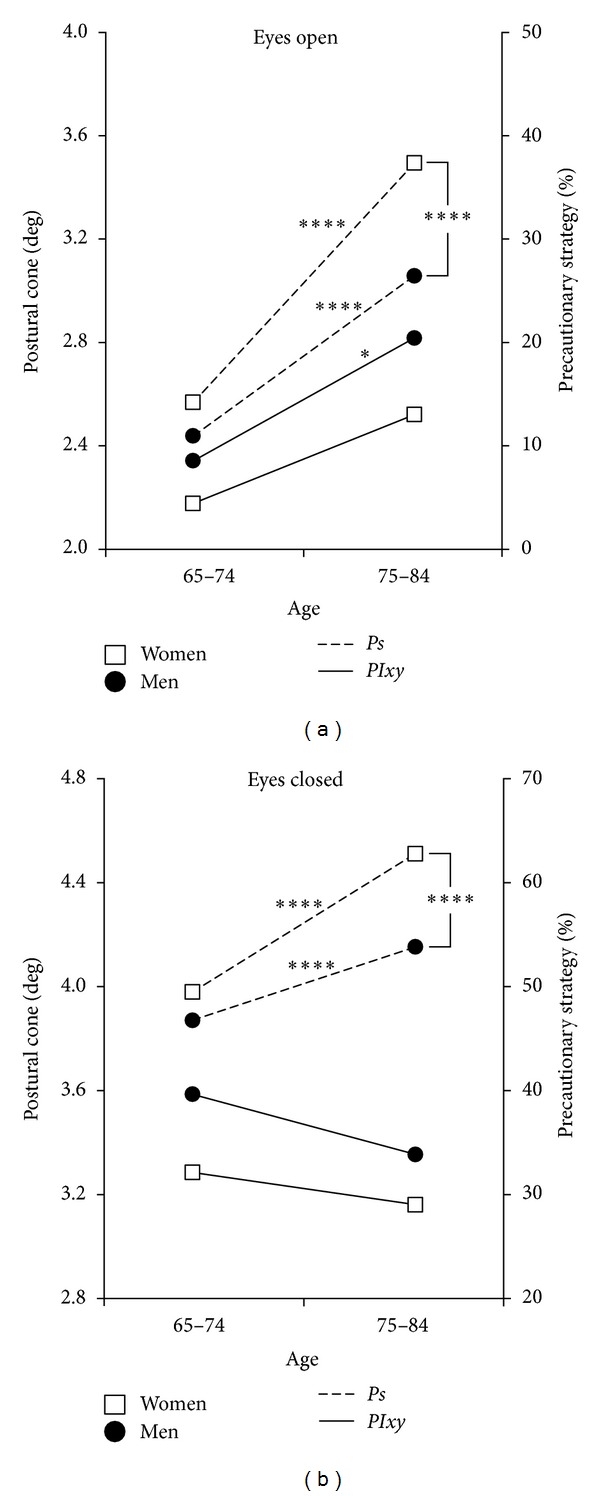
Single stance test: postural cone of instability (*PIxy*) and precautionary strategy (*Ps*) in males and females of different ages. (a) Eyes open. (b) Eyes closed. ****P < 0.001; *P < 0.05.

*Emergency Responses (Tables 2 and 3).* In EO condition (Figure 4(a)) both sexes aged 75–84 enhanced the emergency responses to face the increased instability by widening *PIxy* (*m*: +20.3%, *P* < 0.01; *w*: +15.8%, ns), but females increased the use of *Ps* more than males (*m*: +15.5 pp, *P* < 0.001; *w*: +23.2 pp, *P* < 0.001). In EC trials the situation was different (Figure 4(b)): for both sexes, the older subjects narrowed *PIxy* (*m*: −6.5%, *w*: −3.7%) with respect to the younger individuals and faced the higher instability by significantly increasing *Ps* (*m*: +7.1 pp, *P* < 0.005; *w*: +13.3 pp, *P* < 0.001) and weakening the emergency responses. 

*Double Stance Test.* The results of DT are shown in Table 4. Considering the performances in both visual conditions, 97.6% of subjects showed complete autonomy. As in ST, the variable for the consideration of the autonomy and the postural cone was *SI*. No significant differences emerged between genders in each class of age. In EO condition the subjects aged 65–74 years were significantly more stable than the older ones, both for males (*P* < 0.005) and females (*P* < 0.01). In EC, comparing the younger to the older individuals, the stability decreased significantly both for males (*P* < 0.05) and females (*P* < 0.05). In EO, *PIxy* and *SI* showed a correlation (Pearson) of 0.94 while in EC the correlation was lower (0.67). No significant correlation was present between DT and ST.

## 4. Discussion

The main purpose of the present study was to describe the single stance stability in a representative sample of older subjects living at home, by means of a reliable instrumented test, highlighting and explaining the differences related to age and gender. Moreover, a fundamental goal was to quantify the primary role of proprioception, the compensatory intervention of the visual stabilizer, and the capability of activating the emergency responses. 

### 4.1. Single Stance Stability

Single stance stability significantly worsened with age both in EO and EC conditions, showing significant gender differences in the older class of age. It worsened significantly more in older women than in older men. *SI* would emerge as the most important basic variable because, taking into consideration *Au* and *PIxy,* it is capable of ranking any level of performance. Instead, the timed tests in single stance, not considering the variations of the postural cone, can only measure the time before the subject touches the ground with the nonsupporting foot [35, 38–40].

#### 4.1.1. Postural and Proprioceptive Control

In most cases proprioceptive control appeared inadequate, even in the presence of apparently sufficient postural control (adequate performance in EO trials).

*Age Differences. *The older subjects showed worse stability than the younger individuals (*P* < 0.001 for both sexes). These findings are consistent with previous literature [35, 38–40]; however, the methodology of the present study is able to quantify how this decline in single stance would be due to proprioceptive impairment (*m*: *P* < 0.005; *w*: *P* < 0.001) and to insufficient compensatory intervention of the visual stabilizer (*P* < 0.001 for both sexes). The consequences were a significant compensatory increase of *Ps* and less autonomous and frailer stability. Previous studies, all conducted in double stance, showed that the somatosensory component of postural stability increased its relative importance associated with age [8, 15, 36, 37] while vision reduced its contribution as postural stabilizer in the older subjects [8].

*Gender Differences.* Younger men performed slightly better than women, but the differences were not significant. In the older subjects (75–84 years), instead, women were significantly less stable than men, as a consequence of less effective proprioceptive control (*P* < 0.001) while visual gain was not significantly different between genders (the decrement of visual gain was similar in both sexes between the two classes of age). To our knowledge, age-related differences by gender of the proprioceptive component of single stance stability have not been shown in previous studies. 

#### 4.1.2. Visual Dependence

The compensatory intervention of the visual stabilizer would make the subject only apparently more stable.

In fact, previous studies showed that older fallers had a greater visual dependence than older nonfallers [21, 22]. The necessity of maintaining the eyes anchored to some points of the environment could explain why the stability depending on vision would be limited in range and poorly adaptable. This frail stability would lead to more and more simplified motor tasks. The results showed that very low proprioceptive control would limit partially or completely the visual gain in EO condition as compensatory postural stabilizer (Figure 3(b)). This situation would suggest that a minimum level of proprioceptive control is required to trigger the motor countermeasures activated by the other sensory systems. This hypothesis would be supported by our daily experience: in the subjects with very low proprioceptive control and deactivation of the visual gain, a moderate increase in proprioceptive control reactivates the visual gain, further enhancing single stance stability.

**Figure 5:**
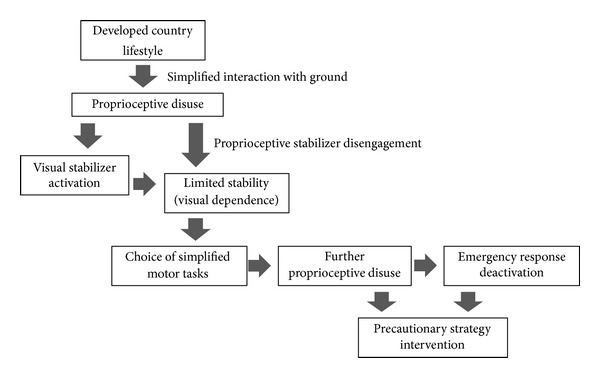
Causal chain of proprioceptive disuse and consequences. The sequence highlights how the intervention of vision as compensatory stabilizer leads to further proprioceptive disengagement.

Some authors considered visual dependence as a response to the unavoidable proprioceptive and vestibular decline resulting from aging and chronic health problems [21, 22]. Other authors, instead, considered partially interchangeable the contribution of the different sensory components as an expression of the plasticity of the sensory systems [15]. Our results would suggest a different scenario where proprioceptive control plays a primary and conditioning role as postural stabilizer. The compensatory intervention of vision as postural stabilizer would be due to the impairment of proprioceptive control mainly because of disuse and could lead to further proprioceptive disengagement and to the deactivation of the emergency responses (Figure 5). Therefore, the primary function of vision should be to allow the best interaction with the environment and to avoid hazards [49]. This approach would not contrast with the studies which suggest that maximizing vision is an effective strategy for preventing falls [20, 50].

#### 4.1.3. Emergency Responses

**Figure 6:**
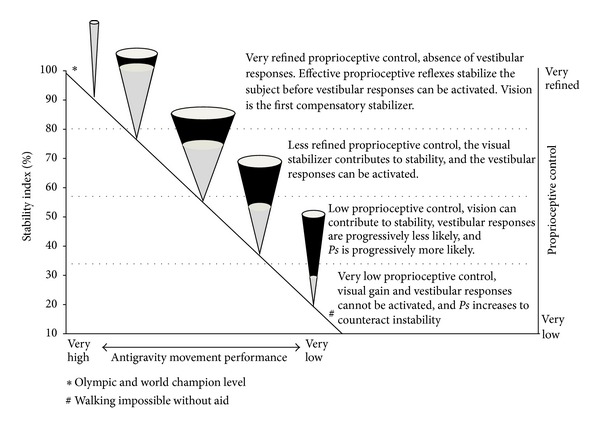
Single stance test (ST): model of interpretation. The impairment of stability is faced first by widening the postural cone (*PIxy*) and then by activating the precautionary strategy (*Ps*, black area of the cone in %). Then, if necessary, *Ps* increases further while *PIxy* tends progressively to become smaller. This behavior is observed both in eyes open and eyes closed (EC) conditions, even if in the latter condition it is more evident. The stability index of EC trials is an indicator of the effectiveness of proprioceptive control, even if the vestibular component is not excluded. From top to bottom higher values of stability index indicate more refined proprioceptive control. Didactically four functional zones can be identified (their dimensions are approximate). At the lower level of stability, the subject is unable to maintain the single stance without continuous hand support and cannot walk without aid. ST levels correspond to different antigravity movement performances.

*Age Differences.* Despite rising instability, the narrowing of *PIxy* for both males and females in the older class of age (in EC) showed a change in the compensatory strategy between younger and older subjects. The results highlighted that the older subjects of both genders, even if more unstable than the younger individuals, presented a narrower *PIxy*, managing the increased instability with greater intervention of *Ps* (Figure 4(b)). To understand this change, the subject in single stance should be described as a broken line with multiple joints, instead of a straight vertical line tilting like an inverse pendulum. The capability of managing the body as a broken line is indispensable for maintaining the projection of the center of gravity in the area of support of the foot. The results would show that this capacity of breaking the line is progressively lost when proprioceptive control is impaired. In fact, subjects with high *Ps* generally present a narrow *PIxy* in EC situation, an expression of their incapacity to activate the emergency responses (Figure 6): they behave as rigid structures and tend to fall like a stick.

#### 4.1.4. Consequences of Aging or Functional and Structural Disuse?

The results suggest that the incapacity to activate compensatory intervention (visual stabilization and emergency responses) would be mostly consequent to the impairment of proprioceptive control. This decline could be firstly the consequence of disuse and only secondly the result of aging [51, 52]. In our opinion, this process would not compromise the potential of the functional reserve of the proprioceptive system. In fact the redundancy of its billions of receptors [53] would make it theoretically less sensitive to the effects of aging. The visual system, instead, is a postural stabilizer with significant anatomical and functional fragilities which favor its exposure to aging [54]. Consequently, postural control heavily based on this system can lead to a dramatic loss of stability in case of its decay. It may be hypothesized that the increasing intervention of the visual system in controlling single stance stability in the younger subjects (65–74 years) could contribute to the disengagement of the proprioceptive stabilizer leading to a more accentuated loss of stability in older age (Figure 5).

#### 4.1.5. Gender Differences in the Older Subjects

The greater deterioration of single stance stability in older females would seem mainly due to more accentuated proprioceptive decay. In fact, the decrease in visual compensation and the reduction in the capacity of activating the emergency responses are similar in both sexes. It could be supposed that the more pronounced proprioceptive decay in women is due both to their muscle weakness which reaches a critical level earlier than in men [47, 55, 56] and to faster proprioceptive disuse (Figure 5) caused by more simplified interaction with the ground, consequent to the kind of shoes worn [57, 58], slower walking speed [47, 59], and so forth. 

### 4.2. Double Stance Stability

Double stance stability significantly worsened with age in both EO and EC conditions but did not show significant gender differences. Age-related differences are consistent with all previous studies. Even if gender differences have been little studied and in small samples, our findings are consistent with most previous studies [36, 37]. The absence of a significant correlation between DT and ST would suggest that DT is not predictive of single stance stability and consequently of the effectiveness of the most critical phase of walking and of the other antigravity movements.

A possible limitation of the study could be the self-selection of the responders/attendees. It is known that the following subgroups tend to be underrepresented among the attendees: males, unmarried and separated/divorced subjects, immigrants, subjects with lower education, and low income receivers of disability benefit [60]. Although sampling methods, selection criteria, data collection, and purposes of the present study should minimize the problem of self-selection, a residual bias in the interpretation of the results could not be excluded completely.

## 5. Conclusions

**Figure 7:**
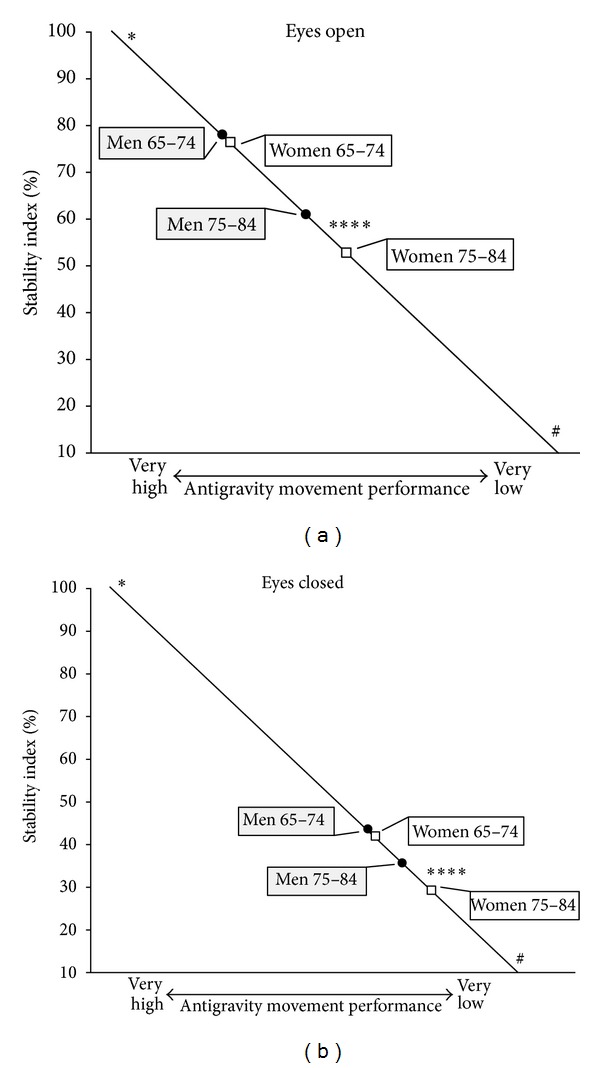
Single (ST). Postural and proprioceptive control: age and gender relations. The age groups of the study are positioned according to their ST level (a) in eyes open (indicator of postural control) and (b) in eyes closed (indicator of proprioceptive control). *Olympic and world champion level; ^#^walking impossible without aid; ****P < 0.001.

The present study pointed out the characteristics of single stance stability in a large sample of older adults, highlighting the differences related to age and gender. Measurements obtained from the single stance test were used to quantify the primary role of proprioceptive control and the compensatory contribution of the visual stabilizer and of the emergency responses. The decline of single stance stability evidenced significant age-related gender differences (Figures 7(a) and 7(b)): while younger women aged 65–74 were not significantly different from men of the same age, women aged 75–84 were significantly less stable than men. This difference would appear to be the consequence of less effective proprioceptive control while the significant decrement in visual gain between the two classes of age was similar in both genders. The stabilizing action of the visual gain in the younger individuals, apparently positive, would guarantee limited and poorly adaptable stability. This frail stability would be a concurrent cause of proprioceptive decay because, leading to simplified motor tasks, it would accentuate proprioceptive disuse (Figure 5). Eyes closed condition was useful for simulating what happens when the visual stabilizer impairs its action or when visibility and luminosity are low in eyes closed: the emergency responses of the older subjects decreased in both sexes with respect to the younger subjects. The study showed that the higher instability of older subjects would be compensated neither by enhancing visual stabilization nor by the emergency responses, but by significantly increasing the precautionary strategy (Figure 6). Thus proprioceptive control would emerge as the real critical element in managing single stance stability. These findings are underlined by the observation that very low proprioceptive control would deactivate the compensatory action of the visual stabilizer and of the emergency responses. Single stance stability based on adequate proprioceptive control would appear to be a promising potential countermeasure which could mitigate the effects of all other intrinsic and extrinsic contributing causes of instability and risk of falling. The findings would suggest that the measurement of the sensory components of single stance stability could be an early predictor of functional decay in walking and antigravity movements, many years before the risk of falling becomes evident. As a development of the study, a follow-up of the participants is being carried out in order to assess the association between single stance stability and health outcomes. Considering that proprioceptive control in single stance would appear to be a key element of the safety and effectiveness of mobility, future research on the possibility of awakening its sleeping potential is suggested.

## Figures and Tables

**Table 1:** Participant characteristics. Values are mean ± SD.

Age	Younger class (65–74)		Older class (75–84)	
Sex	Women	Men	Women	Men
Number of subjects	117	119	161	200
Age (years)	70.0 ± 2.5	69.8 ± 2.5	79.5 ± 2.6	79.4 ± 2.5
Height (cm)	160.3 ± 6.0	171.0 ± 6.4	160.0 ± 5.9	169.7 ± 6.3
Body mass (kg)	65.7 ± 10.6	78.6 ± 10.9	64.0 ± 10.9	75.0 ± 10.8
Body mass index (kg/m^2^)	25.6 ± 4.3	26.9 ± 3.5	25.1 ± 4.5	26.0 ± 3.4
Instruction level				
High	35	57	31	61
Medium	51	38	59	77
Low	31	24	71	62

**Table 2:** The results of the single stance test (ST). Each variable in the first column is identified by a compound name which includes the eye condition and the name of the basic variable.

Variables	Eyes	Unit	Sex	Mean ± SD		*P* level*
				65–74 yrs	75–84 yrs	
EO_Au	Open	%	women	85.8 ± 20.0	62.6 ± 27.3	<0.001
			men	89.0 ± 17.4	73.6 ± 23.1	<0.001
			women	85.8 ± 20.0		ns
			men	89.0 ± 17.4		
			women		62.6 ± 27.3	<0.001
			men		73.6 ± 23.1	

EO_SI	Open	%	women	74.9 ± 20.4	52.7 ± 24.8	<0.001
			men	76.6 ± 17.5	60.8 ± 21.7	<0.001
			women	74.9 ± 20.4		ns
			men	76.6 ± 17.5		
			women		52.7 ± 24.8	<0.001
			men		60.8 ± 21.7	

EC_Au	Closed	%	women	50.5 ± 17.9	37.2 ± 19.0	<0.001
			men	53.2 ± 19.2	46.2 ± 21.2	<0.005
			women	50.5 ± 17.9		ns
			men	53.2 ± 19.2		
			women		37.2 ± 19.0	<0.001
			men		46.2 ± 21.2	

EC_SI	Closed	%	women	40.0 ± 14.6	29.0 ± 15.2	<0.001
			men	41.4 ± 15.8	35.7 ± 17.2	<0.005
			women	40.0 ± 14.6		ns
			men	41.4 ± 15.8		
			women		29.0 ± 15.2	<0.001
			men		35.7 ± 17.2	

EO: Eyes open; EC: Eyes closed; SI: Stability index; Au: Autonomy. The values are calculated considering the average between the two legs. *significance level according to *t*-test.

**Table 3:** The visual gain and the emergency responses in the single stance test (ST). Each variable in the first column is identified by a compound name which includes the eye condition and the name of the basic variable.

Variables	Eyes	Unit	Sex	Mean ± SD		*P* level*
				65–74 yrs	75–84 yrs	
Visual gain (EO-EC)_SI	Open closed	pp	Women	34.8 ± 15.7	23.8 ± 17.6	<0.001
			Men	35.2 ± 16.0	25.1 ± 17.4	<0.001
			Women	34.8 ± 15.7		ns
			Men	35.2 ± 16.0		
			Women		23.8 ± 17.6	ns
			Men		25.1 ± 17.4	

Emergency responses EO_PIxy	Open	Degrees	Women	2.2 ± 1.7	2.5 ± 1.2	ns
			Men	2.3 ± 1.6	2.8 ± 1.6	<0.05

Emergency responses EC_PIxy	Closed	Degrees	Women	3.3 ± 1.5	3.2 ± 1.3	ns
			Men	3.6 ± 1.6	3.4 ± 1.8	ns

EO: Eyes open; EC: Eyes closed; SI: Stability index; PIxy: Postural cone amplitude; pp: Percentage points. The values are calculated considering the worse leg in the EC trials. *significance level according to *t*-test.

**Table 4:** The results of the Double Stance Test (DT). Each variable in the first column is identified by a compound name which includes the eye condition and the name of the basic variable.

Variables	Eyes	Unit	Sex	Mean ± SD		*P* level*
				65–74 yrs	75–84 yrs	
EO_SI	Open	%	Women	95.87 ± 1.76	95.23 ± 1.95	<0.01
			Men	95.83 ± 1.20	95.41 ± 1.28	<0.005

EC_SI	Closed	%	Women	94.28 ± 6.00	92.79 ± 4.94	<0.05
			Men	94.23 ± 1.64	93.28 ± 4.04	<0.05

EO_PIxy	Open	Degrees	Women	0.45 ± 0.14	0.54 ± 0.22	<0.001
			Men	0.46 ± 0.13	0.51 ± 0.15	<0.005

EC_PIxy	Closed	Degrees	Women	0.58 ± 0.17	0.78 ± 0.47	<0.001
			Men	0.65 ± 0.19	0.73 ± 0.26	<0.01

EO: Eyes open; EC: Eyes closed; SI: Stability index; PIxy: Postural cone amplitude. The best trial, in the same eye condition, is considered. *significance level according to *t*-test.

## Abbreviations

*Au*: Autonomy
EC: Eyes closed
*DVC*: Delos vertical controller
*dDVC*: Absolute instant deviation of DVC measure from the average axis
DT: Double stance test
EO: Eyes open
*PIx*: Average postural instability in the frontal plane
*PIxy*: Average postural instability or amplitude of the postural cone
*PIy*: Average postural instability in the sagittal plane
*Ps*: Precautionary strategy (the complementary value of *Au*)
*SI*: Stability index
*spv*: Sample performance value
ST: Single stance test.

## References

[1] Todd C, Skelton D (2004). What are the main risk factors for falls among older people and what are the most effective interventions to prevent these falls?. *Health Evidence Network Report*.

[2] Heinrich S, Rapp K, Rissmann U, Becker C, König H-H (2010). Cost of falls in old age: a systematic review. *Osteoporosis International*.

[3] Masud T, Morris RO (2001). Epidemiology of falls. *Age and Ageing*.

[4] Guralnik JM, Ferrucci L, Simonsick EM, Salive ME, Wallace RB (1995). Lower-extremity function in persons over the age of 70 years as a predictor of subsequent disability. *New England Journal of Medicine*.

[5] Studenski S, Perera S, Wallace D (2003). Physical performance measures in the clinical setting. *Journal of the American Geriatrics Society*.

[6] Montero-Odasso M, Schapira M, Soriano ER (2005). Gait velocity as a single predictor of adverse events in healthy seniors aged 75 years and older. *Journals of Gerontology A*.

[7] Nashner LM, Black FO, Wall C (1982). Adaptation to altered support and visual conditions during stance: patients with vestibular deficits. *Journal of Neuroscience*.

[8] Lord SR, Ward JA (1994). Age-associated differences in sensori-motor function and balance in community dwelling women. *Age and Ageing*.

[9] Lord SR, Lloyd DG, Li SK (1996). Sensori-motor function, gait patterns and falls in community-dwelling women. *Age and Ageing*.

[10] Kobayashi H, Hayashi Y, Higashino K (2002). Dynamic and static subjective visual vertical with aging. *Auris Nasus Larynx*.

[11] Callisaya ML, Blizzard L, McGinley JL, Schmidt MD, Srikanth VK (2010). Sensorimotor factors affecting gait variability in older people-a population-based study. *Journals of Gerontology A*.

[12] Sherrington CS (1906). *The Integrative Action of the Nervous System*.

[13] Riemann BL, Lephart SM (2002). The sensorimotor system, part I: the physiologic basis of functional joint stability. *Journal of Athletic Training*.

[14] Moruzzi G (1975). *Physiology of Relational Life*.

[15] Cohen H, Heaton LG, Congdon SL, Jenkins HA (1996). Changes in sensory organization test scores with age. *Age and Ageing*.

[16] Lord SR (2006). Visual risk factors for falls in older people. *Age and Ageing*.

[17] Coleman AL, Cummings SR, Yu F (2007). Binocular visual-field loss increases the risk of future falls in older white women. *Journal of the American Geriatrics Society*.

[18] Freeman EE, Broman AT, Turano KA, West SK (2008). Motion-detection threshold and measures of balance in older adults: the SEE project. *Investigative Ophthalmology and Visual Science*.

[19] Salonen L, Kivelä SL (2012). Eye diseased and impaired vision as possible risk factors for recurrent falls in the aged: a systematic review. *Current Gerontology and Geriatrics Research*.

[20] Lord SR, Smith ST, Menant JC (2010). Vision and falls in older people: risk factors and intervention strategies. *Clinics in Geriatric Medicine*.

[21] Tobis JS, Reinsch S, Swanson JM (1985). Visual perception dominance of fallers among community-dwelling older adults. *Journal of the American Geriatrics Society*.

[22] Lord SR, Webster IW (1990). Visual field dependence in elderly fallers and non-fallers. *International Journal of Aging and Human Development*.

[23] Murray MP, Drought AB, Kory RC (1964). Walking patterns of normal men. *The Journal of Bone and Joint Surgery*.

[24] Rodgers MM (1988). Dynamic biomechanics of the normal foot and ankle during walking and running. *Physical Therapy*.

[25] Winter DA (2009). *Biomechanics and Motor Control of Human Movement*.

[26] Murray MP, Kory RC, Clarkson BH (1969). Walking patterns in healthy old men. *Journals of Gerontology*.

[27] Elble RJ, Thomas SS, Higgins C, Colliver J (1991). Stride-dependent changes in gait of older people. *Journal of Neurology*.

[28] Woo J, Ho SC, Lau J, Chan SG, Yuen YK (1995). Age-associated gait changes in the elderly: pathological or physiological?. *Neuroepidemiology*.

[29] Chamberlin ME, Fulwider BD, Sanders SL, Medeiros JM (2005). Does fear of falling influence spatial and temporal gait parameters in elderly persons beyond changes associated with normal aging?. *Journals of Gerontology A*.

[30] Maki B (1997). Gait changes in older adults: predictors of falls or indicators of fear?. *Journal of the American Geriatrics Society*.

[31] Hausdorff JM, Levy BR, Wei JY (1999). The power of ageism on physical function of older persons: reversibility of age-related gait changes. *Journal of the American Geriatrics Society*.

[32] Menz HB, Lord SR, Fitzpatrick RC (2003). Age-related differences in walking stability. *Age and Ageing*.

[33] Riva D, Rossitto F, Battocchio L (2009). Postural muscle atrophy prevention and recovery and bone remodelling through high frequency proprioception for astronauts. *Acta Astronautica*.

[34] Overstall PW, Exton Smith AN, Imms FJ, Johnson AL (1977). Falls in the elderly related to postural imbalance. *British Medical Journal*.

[35] Bohannon RW, Larkin PA, Cook AC (1984). Decrease in timed balance test scores in aging. *Physical Therapy*.

[36] Colledge NR, Cantley P, Peaston I, Brash H, Lewis S, Wilson JA (1994). Ageing and balance: the measurement of spontaneous sway by posturography. *Gerontology*.

[37] Hageman PA, Leibowitz JM, Blanke D (1995). Age and gender effects on postural control measures. *Archives of Physical Medicine and Rehabilitation*.

[38] Balogun JA, Akindele KA, Nihinlola J, Marzouk DK (1994). Age-related changes in balance performance. *Disability and Rehabilitation*.

[39] Morioka S, Fukumoto T, Hiyamizu M, Matsuo A, Takebayashi H, Miyamoto K (2012). Changes in the equilibrium of standing on one leg at various life stages. *Current Gerontology and Geriatrics Research*.

[40] Springer BA, Marin R, Cyhan T, Roberts H, Gill NW (2007). Normative values for the unipedal stance test with eyes open and closed. *Journal of Geriatric Physical Therapy*.

[41] Wolfson L, Whipple R, Derby CA, Amerman P, Nashner L (1994). Gender differences in the balance of healthy elderly as demonstrated by dynamic posturography. *Journals of Gerontology*.

[42] Cardano M, Costa G, Demaria M (2004). Social mobility and health in the Turin longitudinal study. *Social Science and Medicine*.

[43] Delos Postural Proprioceptive System. http://www.delos-international.com/prodotti.asp?sec=scheda&lang=eng.

[44] Baldissera F, Bertocchi G, Cavalari P (1999). *Neurosciences*.

[45] Bacsi AM, Colebatch JG (2005). Evidence for reflex and perceptual vestibular contributions to postural control. *Experimental Brain Research*.

[46] Simonsick EM, Newman AB, Visser M (2008). Mobility limitation in self-described well-functioning older adults: importance of endurance walk testing. *Journals of Gerontology A*.

[47] Cooper R, Hardy R, Sayer A (2011). Age and gender differences in physical capability levels from mid-life onwards: the harmonisation and meta-analysis of data from eight UK cohort studies. *PLoS ONE*.

[48] Rousson V, Gasser T, Seifert B (2002). Assessing intrarater, interrater and test-retest reliability of continuous measurements. *Statistics in Medicine*.

[49] Campbell AJ, Borrie MJ, Spears GF, Jackson SL, Brown JS, Fitzgerald JL (1990). Circumstances and consequences of falls experienced by a community population 70 years and over during a prospective study. *Age and Ageing*.

[50] de Boer MR, Pluijm SMF, Lips P (2004). Different aspects of visual impairment as risk factors for falls and fractures in older men and women. *Journal of Bone and Mineral Research*.

[51] Hutton RS, Atwater SW (1992). Acute and chronic adaptations of muscle proprioceptors in response to increased use. *Sports Medicine*.

[52] Shaffer SW, Harrison AL (2007). Aging of the somatosensory system: a translational perspective. *Physical Therapy*.

[53] Ian McCloskey D (1994). Human proprioceptive sensation. *Journal of Clinical Neuroscience*.

[54] Klein R (1991). Age-related eye disease, visual impairment, and driving in the elderly. *Human Factors*.

[55] Frontera WR, Hughes VA, Lutz KJ, Evans WJ (1991). A cross-sectional study of muscle strength and mass in 45- to 78-yr-old men and women. *Journal of Applied Physiology*.

[56] Campbell AJ, Borrie MJ, Spears GF (1989). Risk factors for falls in a community-based prospective study of people of 70 years and older. *Journals of Gerontology*.

[57] Menant JC, Steele JR, Menz HB, Munro BJ, Lord SR (2008). Effects of footwear features on balance and stepping in older people. *Gerontology*.

[58] Cronin NJ, Barrett RS, Carty CP (2012). Long-term use of high-heeled shoes alters the neuromechanics of human walking. *Journal of Applied Physiology*.

[59] Butler AA, Menant JC, Tiedemann AC (2009). Age and gender differences in seven tests of functional mobility. *Journal of NeuroEngineering and Rehabilitation*.

[60] Søgaard AJ, Selmer R, Bjertness E, Thelle D (2004). The Oslo health study: the impact of self-selection in a large, population-based survey. *International Journal for Equity in Health*.

